# Early mobility to improVE health outcomes following cardiac surgery (EVE) clinical trial development: findings from workshop 2—co-created definition and prioritized barriers, facilitators and outcome and experience measures

**DOI:** 10.3389/fresc.2026.1801097

**Published:** 2026-10-08

**Authors:** Emily K. Phillips, Jacqueline L. Hay, April Gregora, Kathy Smith, Anna M. Chudyk, Carly Shaski, Sarah Gilchrist, Andrew Fagan, Maureen C. Ashe, Annette S. H. Schultz, Rakesh C. Arora, Todd A. Duhamel, Sheila O’Keefe-McCarthy

**Affiliations:** 1 Applied Health Sciences, Faculty of Graduate Studies, University of Manitoba, Winnipeg, MB, Canada; 2 Faculty of Kinesiology and Recreation Management, University of Manitoba, Winnipeg, MB, Canada; 3 Institute of Cardiovascular Sciences, St. Boniface Hospital Albrechtsen Research Centre, Winnipeg, MB, Canada; 4 Person with Lived Experience Partner, University of Manitoba, Winnipeg, MB, Canada; 5 College of Pharmacy, Rady Faculty of Health Sciences, University of Manitoba, Winnipeg, MB, Canada; 6 St. Boniface Hospital, Winnipeg, MB, Canada; 7 Max Rady College of Medicine, Rady Faculty of Health Sciences, University of Manitoba, Winnipeg, MB, Canada; 8 Department of Family Practice, The University of British Columbia, Vancouver, BC, Canada; 9 College of Nursing, Rady Faculty of Health Sciences, University of Manitoba, Winnipeg, MB, Canada; 10 Department of Surgery, Northwestern University, Feinberg School of Medicine, Chicago, IL, United States; 11 School of Nursing, Faculty of Applied Health Sciences, Brock University, St. Catharines, ON, Canada

**Keywords:** cardiac surgery, intensive care, post-intensive care syndrome, PICS, early mobility, patient and public involvement, patient engagement in research

## Abstract

**Introduction:**

New or worsening cognitive, mental, and/or physical health after a stay in an intensive care unit (ICU) defines post-intensive care syndrome (PICS). PICS is prevalent post-cardiac surgery (CS), and interventions are needed for prevention. Early mobility shows potential to prevent PICS but is not optimally integrated into CSICU clinical care. This may relate to the absence of engagement of people with lived experience (PWLE) in CSICU early mobility research.

**Methods:**

PWLE of the CSICU were engaged to meet three objectives: 1) co-create a definition of early mobility, 2) identify barriers and facilitators to early mobility, and 3) prioritize outcome and experience measures for use in future intervention research. Engagement occurred through two virtual consultation workshops involving group discussions, consensus building, and prioritization using the nominal group technique.

**Results:**

Eight patients and two care partners participated. The co-created definition of early mobility was *a progression of functional life activities that begins as soon as the patient can participate (typically within six hours post-surgery), where the patient uses their muscle strength intentionally.* The top barrier to early mobility was brain “fuzziness/fogginess”. The top facilitator was a “positive attitude”. The top physical outcome was “Do you feel lightheaded/dizzy?”. The top cognitive outcome was “Do you feel fuzzy/foggy”. The top mental outcome was “Are you focusing on the positives in your life right now?”. The top experience measure was “Do you feel your doctors gave you the care and attention you needed?”.

**Discussion:**

This important contribution of the lived experience with early mobility in the CSICU advances the literature and will empower future research to align with the perspectives and priorities of people undergoing CS. This contribution may advance the integration of early mobility into CSICU clinical care and reduce the prevalence of adverse cognitive, mental, and/or physical health outcomes that are seen in PICS.

## 1 Introduction

Early mobility through ambulation and cycling improves physical health outcomes following cardiac surgery (CS) (1–3). Physical health outcomes are one element of post-intensive care syndrome (PICS), a syndrome defined as a new or worsening deterioration in cognitive, mental, and/or physical health after admission to the intensive care unit (ICU) (4). Our group previously investigated the incidence of PICS following CS, which ranged from 16% to 99% (5). This broad range in incidence reflects the many symptoms that make up each domain of PICS and the diversity in tools used to measure these domains (5). With this potentially high prevalence, interventions are needed to prevent the occurrence of PICS and support optimal health outcomes for patients with a prolonged stay in the CSICU.

Despite best-practice support, integration of early mobility into CSICU clinical care remains underutilized (6, 7). It has been hypothesized that the lack of integration may relate to an absence of engagement of people who have had CS in the past and have experienced early mobility in the CSICU (8, 9). Referred to as patient engagement in research (PER), people with lived experience (PWLE) of a health issue (e.g., patients and their care partners) can be meaningfully and actively engaged in the research process from design to knowledge translation (9–11). Generating knowledge through PER is recognized as a means to improve the healthcare system (9–11). However, there is a lack of PWLE perspectives informing early mobility CSICU literature. For example, there is minimal data on a PWLE-informed definition of early mobility. Similarly, identification of barriers and facilitators that need to be overcome or leveraged to integrate early mobility into CSICU clinical care successfully using a PER approach. Finally, there are a lack of located studies exploring outcome measures important to PWLE when evaluating early mobility interventions. This paucity of PWLE-informed research limits the evidence available to improve the delivery of healthcare through integrating beneficial early mobility interventions informed by PWLE.

This third article in a series of three describes Phase 2 of a two-phased workshop to develop a PWLE-informed early mobility intervention for use in the CSICU. The methods employed in this workshop have been described as a model in the first article (12). The findings of Phase 1, in which the arts-based research method of body mapping (13–17) was used to co-create and co-analyze an understanding of the embodied experience of mobility in the CSICU from PWLE perspectives, have been reported in the second article (18). Phase 2, reported here, employed two virtual consultation workshops to engage PWLE of the CSICU to meet three objectives: 1) to co-create a definition of early mobility, 2) to identify barriers and facilitators to early mobility, and 3) to prioritize outcome and experience measures for use in future intervention research. This work is part of the Early mobility to improVE health outcomes following cardiac surgery (EVE) Clinical Feasibility Trial Development study.

## 2 Methods

### 2.1 Research design

PWLE engagement occurred through two virtual consultation workshops involving group discussions, consensus building, and prioritization using the nominal group technique. Reporting of this work follows the updated Guidelines for Reporting Involvement of Patients and the Public (GRIPP-2) long-form checklist (19). See the supplemental materials for the GRIPP-2 checklist.

### 2.2 PWLE recruitment

The PWLE co-authors (AG & KS) suggested the inclusion of care partners due to one's difficulty in remembering her own CSICU hospitalization (AG), and one's experience as a caregiver for her husband during his CSICU hospitalization (KS). This decision is supported by the literature, which states that care partners play an integral role in organizing memories of the time spent in the CSICU (20)*.* 10 PWLE were recruited to participate in this study. This sample size aligns with PER and nominal group technique best practices and was selected to facilitate discussion and participation and allows for a variety of perspectives (21–27).

A database of patients who have undergone CS at St. Boniface Hospital in Winnipeg, MB, who agreed to be contacted for future research, was used to recruit PWLE. Inclusion criteria were as follows: 1) adults over 18 who have undergone or had a loved one undergo elective or semi-urgent outpatient coronary artery bypass grafting, valve CS, or a combination of the two in the last five years, 2) cognitively able to participate in consultation workshop, 3) had access to the internet and an electronic device, 4) were willing to reflect on personal experiences relating to CS and share these in a group setting, 5) could speak and read English, and 6) consent to be recorded.

### 2.3 Equity, diversity, and inclusion considerations

The diversity of PWLE engaged in this study was limited by the small group size and existing diversity of former CS patients who consented to be approached about future research, who met our inclusion criteria. Due to these limitations, we focused on recruiting PWLE based on diversity at three levels: 1) self-identified gender, 2) patients and care partners, and 3) rural and urban geographic location of participants. Our approach lacks focus on racial and ethnic diversity, which is a limitation. Future research should focus on recruiting diverse groups in both meaningful and culturally sensitive ways that facilitate developing an understanding of the needs of PWLE of different ethnicities, rather than comparing experiences among ethnicities. Inclusion was supported through confidential, personalized discussions with each participant. Any required supports for participation in the virtual workshops were identified. Additionally, we offered two times to meet with PWLE on Microsoft Teams to introduce them to the virtual platform prior to the first virtual workshop session. In this way, we supported equitable access to the virtual workshop sessions to promote participation and comfort with the virtual platform.

### 2.4 Levels and stages of engagement

The International Association for Public Participation's Spectrum of Public Participation (28) was used to define levels of engagement. Engagement is defined along the Spectrum from “inform” to “empower”, with the latter having more impact on research decision-making (28). Similar to other work by the research team (29–31), a multi-level approach to engagement was used. First, two PWLE (AG and KS) were engaged at the level of collaborate (28). This level is in the middle of the Spectrum and is described as “to partner with the public in each aspect of the decision, including the development of alternatives and the identification of the preferred solution” (28). Both PWLE collaborators are members of the research team and contribute lived expertise at all stages of the research through planning meetings, developing the workshop plan, co-facilitating the workshops, co-analyzing the workshop findings, and co-authoring all publications (32). Second, additional PWLE (*n* = 10) were engaged at the level of consult through the PWLE engagement panel, a level described as gaining public feedback to drive research decisions (28). The PWLE consultants were engaged at the stages of data collection and co-analysis (32). Specifically, PWLE consultants were engaged in discussions that generated the data that was used to guide the larger research project. They generated the early mobility definition, barriers, facilitators, and outcome and experience measures through facilitated discussions. They then co-analyzed their data through facilitated prioritization and consensus-building. All words and terms presented in the results were generated and defined by the PWLE consultants. Multi-level engagement allowed this research to be directly guided by lived experiences. Collaborating with PWLE ensured they guided the research process, and consulting additional PWLE ensured a broader range of perspectives contributed to generating the early mobility definition, as well as generating and prioritizing barriers, facilitators, and outcome and experience measures. The levels of engagement informed the compensation provided (33). Compensation is described for both phases of this study in the article describing Phase 1 (34).

### 2.5 Ethical considerations

This work was conducted in alignment with the Tri-Council Policy Statement: Ethical Conduct for Research Involving Humans—TCPS 2 (35). Institutional review board approvals for the case study exemplar were obtained in March 2024 from the University of Manitoba Research Ethics Board 1 (HE2024-0035) and the St. Boniface Hospital Research Review Committee (RRC/2024/2158). A consent form was provided at the time of recruitment for PWLE to review. This consent form was reviewed and signed by all PWLE prior to participating in the workshops. The ongoing nature of consent was discussed with all PWLE. They were made aware that they could leave the workshops at any time and could withdraw consent at any point until data analysis began, at which point it was no longer possible to remove their data from the study. No PWLE withdrew their consent. Demographics were collected using a self-report questionnaire during Workshop 1, including self-identified sex, gender, and ethnicity, role (patient or care partner), ICU length of stay, type of CS, and year of CS.

### 2.6 Approach and data analysis

The CIHR SPOR Framework (9) and previous virtual consultation workshops completed by research team members (21, 36) informed the approach to engaging PWLE. Two virtual workshops were held in June 2024. Data collection and analysis occurred simultaneously throughout the workshops. The aims of the first virtual workshop were to co-create a PWLE-informed definition of early mobility and to generate a list of barriers and facilitators to early mobility and a list of outcome and experience measures to evaluate a future early mobility intervention. The aim of the second virtual workshop was to prioritize the lists of barriers, facilitators, outcome, and experience measures using the nominal group technique.

The engagement approach has been shared in detail elsewhere (12). Briefly, in the first virtual workshop, PWLE discussed what early mobility meant to them to co-create a definition. Next, barriers and facilitators to early mobility were generated and discussed by PWLE. Finally, outcomes and experiences related to early mobility, important to PWLE, were generated and discussed. Following this workshop, the barriers, facilitators, and outcome and experience measures were entered into a survey using Microsoft Forms. The outcome measures were grouped by the first author (EKP) under the three domains of PICS: cognitive, mental, and physical (4, 5). In the second virtual workshop, the barriers, facilitators and outcome and experience measures were prioritized by PWLE using the nominal group technique (21–26). Two rounds of prioritization occurred, with a discussion between rounds focused on the initial prioritization from least agreement to most agreement (21–26). During this discussion, the first author (EKP) revised the survey to capture all changes, such as rephrased or combined research directions. Once the second survey was completed, PWLE logged out of the virtual workshop.

PWLE demographics were described using the median and interquartile range (IQR) for continuous variables and frequencies and proportions for categorical factors (37). Microsoft Forms (version 2) and Excel (version 16.99.2) were used to analyze the second survey responses from PWLE. Where PWLE ranked each item (i.e., first choice, second choice) was noted along with the amount of agreement or disagreement (i.e., items that were both first and last choice by PWLE). The barriers, facilitators, and outcome and experience measures were ranked from most to least agreement based on where each PWLE ranked each item by Microsoft Forms. The top three barriers, facilitators, and outcome measures from each domain of PICS, and experience measures were identified from this ranking. When generated by PWLE, they were stated as questions. They have been rephrased from questions to reflect the underlying concept of interest.

Finally, the definition of early mobility was enhanced by members of the research team after the virtual consultation workshops. This occurred through discussions at research team meetings, written feedback, and discussions with the PWLE and clinician members of the research team. The enhancement of the early mobility definition aimed to ensure clinical utility, as clinicians identified elements missing from the PWLE definition that were necessary. Importantly, no PWLE-identified elements were removed by clinicians; only additions occurred.

## 3 Results

Eight patients and two care partners participated in this study. Four PWLE self-identified their sex/gender as female/women and six as male/men. The PWLE included one male/man care partner, one female/woman care partner, three female/woman patients, and five male/man patients. Demographic information is presented in Table 1. Two people self-identified as Métis, of which one also self-identified as Ojibwe. Two PWLE declined to self-identify their ethnicity. The median age of PWLE was 69 (IQR 61-82) years. The median CSICU length of stay was 5 (IQR 3-30) days. One PWLE did not remember their CSICU length of stay. One PWLE had CS in 2021, two in 2022, and the remaining seven underwent CS in 2023.

**Table 1:** Demographics of PWLE participants.

Participant ID	Age	Sex assigned at birth	Self-identified Gender	Self-identified ethnicity	Role (Patient or care partner)	ICU LOS	Type of CS	Year of CS
P1	82	Male	Man	Irish	Patient	5 days	CABG + Valve	2021
P2	68	Male	Man	n/a	Patient	Unknown???	Valve	2022
P4	60	Female	Woman	White	Patient	6 days	CABG + Valve	2023
P5	63	Male	Man	Metis	Care Partner	6 days	CABG + Valve	2023
P6	70	Male	Man	Participant left blank	Patient	30 days	CABG	2023
P7	59	Female	Woman	Caucasian	Patient	3 days	Valve	2022
P8	80	Female	Woman	Canadian-French	Patient	7 days	valve	2023
P9	75	Male	Man	White/Caucasian	Patient	3 days	CABG	2023
P10	73	Male	Man	CDN	Patient	5 days	CABG	2023
P11	60	Female	Woman	Metis/Ojibwe	Care Partner	3 days	CABG	2023

### 3.1 Definition of early mobility

Initially, PWLE shared a variety of definitions of “*early*” and “*mobility*”. Definitions of *early* ranged from the “day of surgery” to “discharge from the ICU” (clarified to mean after ICU discharge). After a discussion, PWLE came to the consensus that the definition of early needs to be person-specific and tied to patient assessments and meeting individual patient-defined milestones rather than a specific timeframe. *Mobility* was initially defined as sitting and standing at the side of the bed, sitting in a chair, and walking. After a discussion, PWLE came to the consensus that mobility needs to be defined in terms of a spectrum of activities that will achieve the ultimate goal of getting home, as well as how patients need to move to accomplish this. This led to the PWLE definition of early mobility, encompassing a spectrum of activities required for daily living, that begin when a patient in the ICU is ready to engage in activities following CS. This PWLE definition was enhanced by the clinician team members as described above. The final co-created definition of early mobility was *a progression of functional life activities that begins as soon as the patient can participate (typically within six hours post-surgery), where the patient uses their muscle strength intentionally.*

### 3.2 Prioritization of barriers, facilitators of early mobility

The results of the prioritization are presented in Figures 1,2. The top three barriers to early mobility were brain “fuzziness” or “fogginess”, “paraphernalia” (lines, tubes, etc.), and “weakness” (Figure 1). The top three facilitators of early mobility were a “positive attitude”, “being encouraged/ forced/ helped/ reminded”, and “receiving education about pre-existing health issues interacting and causing unclear discomfort” (Figure 2).

**Figure 1:**
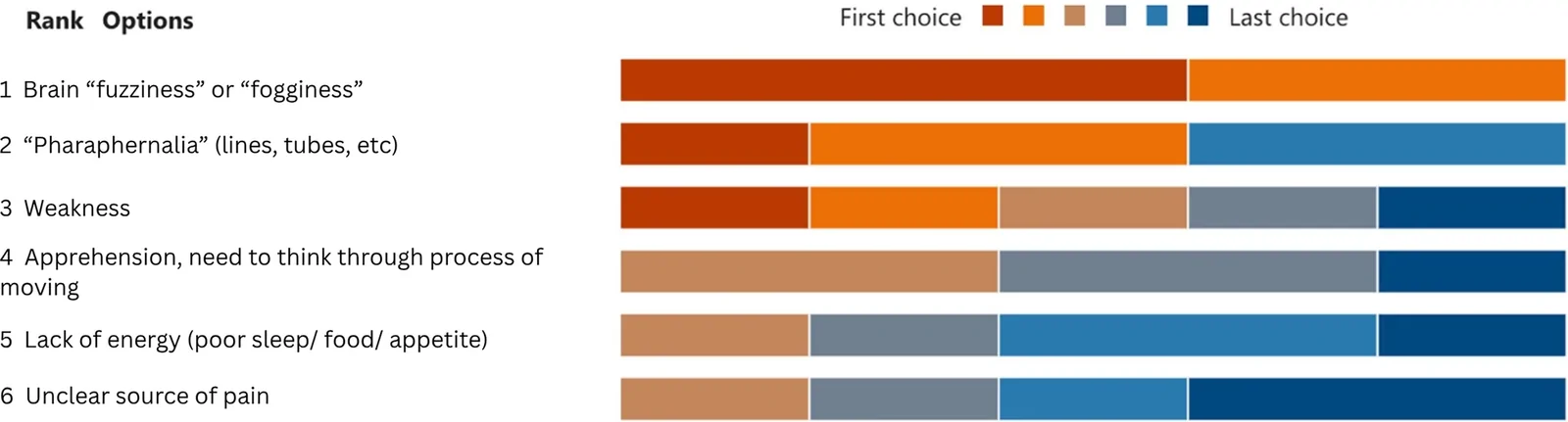
Ranking of barriers to early mobility in the CSICU.

**Figure 2:**
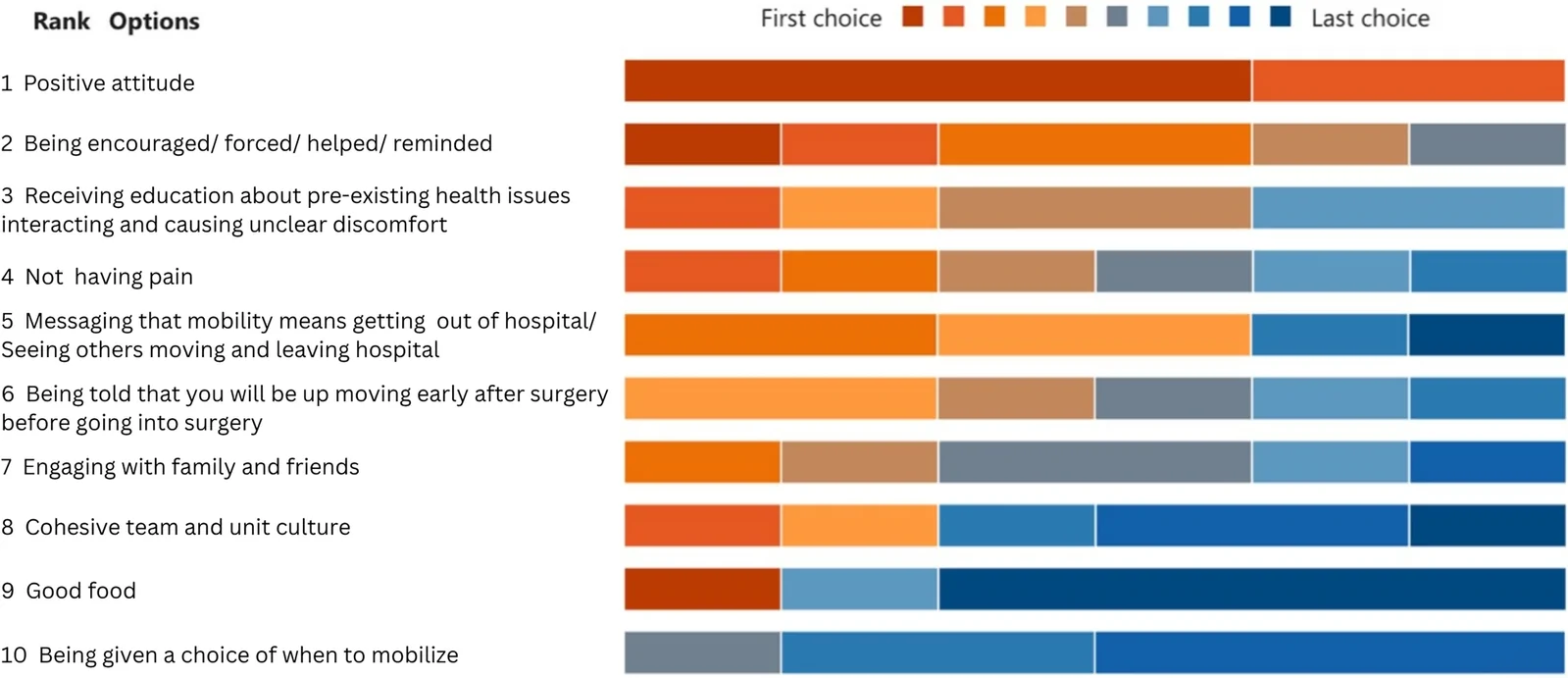
Ranking of facilitators to early mobility in the CSICU.

### 3.3 Prioritization of outcome measures and experience measures of early mobility

Outcome measure concepts for use in future intervention research were divided into physical, cognitive, and mental outcomes to support their future use in PICS research (Table 2, Figures 3–6). The top three physical outcomes were light-headedness/ dizziness, balance, and confidence with mobility (Figure 3). The top three cognitive outcomes were head fuzziness/ fogginess, visual hallucinations, and perceived confusion or visual hallucinations (Figure 4). The top three mental outcomes were a positive outlook, anxiety, and a negative outlook (Figure 5). The top three experience measures were “Do you feel your doctors gave you the care and attention you needed?”, “Do you feel your nurses gave you the care and attention you needed?”, and “Do you feel the care team asked how you were doing and listened to your answer?” (Figure 6).

**Table 2:** Outcome measures generated by people with lived experience of early mobility in the cardiac surgery intensive care unit.

Domain	Original questions generated by people with lived experience	Rephrased outcome measure
Physical	Do you feel lightheaded or dizzy?	Light-headedness/ dizziness
	Do you feel you have balance when you stand and walk?	Balance
	Do you have confidence in moving?	Confidence with mobility
Cognitive	Do you feel fuzzy/ foggy?	Head fuzziness/ fogginess
	Are you seeing anything strange/ odd/ hallucinating?	Visual hallucinations
	Care partner question—does your loved one seem confused/ are they hallucinating?	Perceived confusion or visual hallucinations
Mental	Are you focusing on the positives in your life right now?	Positive outlook
	Are you feeling anxious?	Anxiety
	Are you focusing on the negatives in your life right now?	Negative outlook

**Figure 3:**
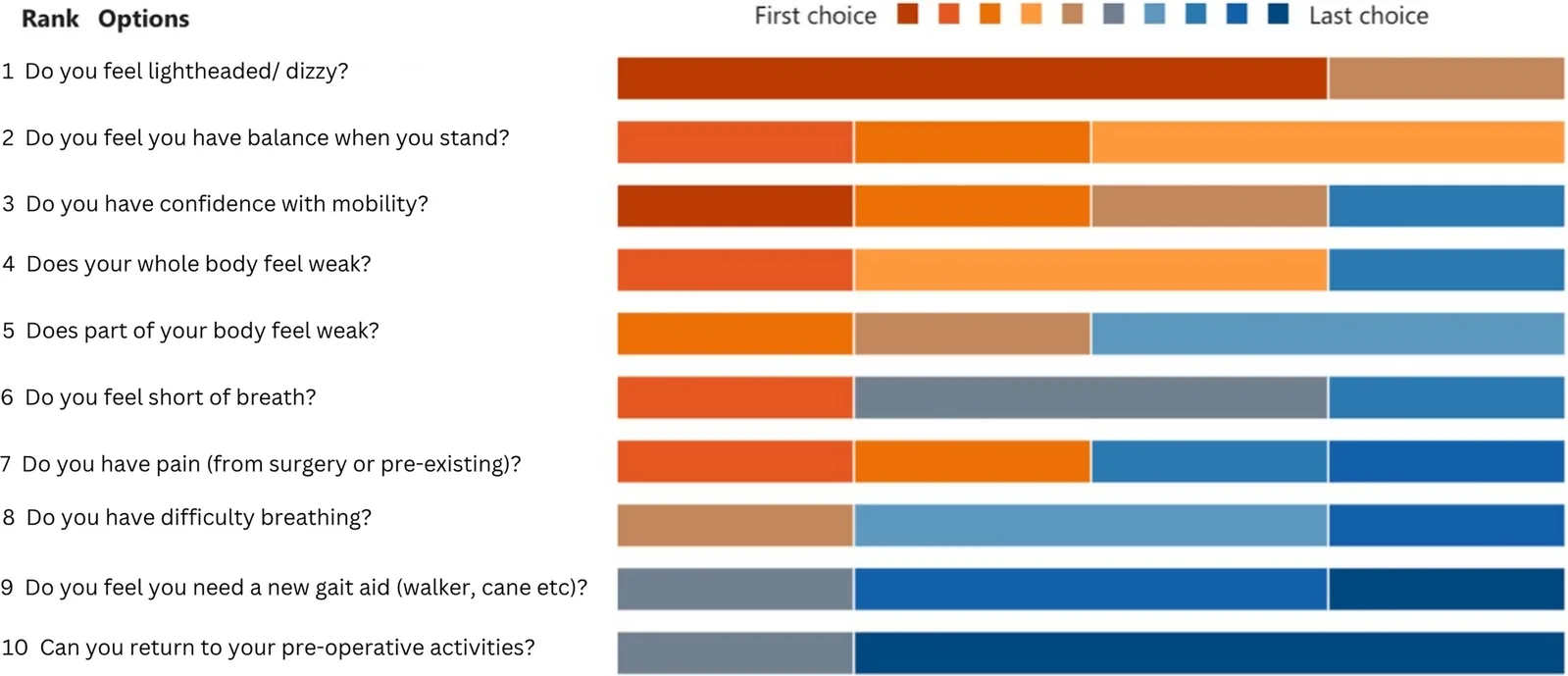
Ranking of the physical outcome measures of early mobility in the CSICU.

**Figure 4:**
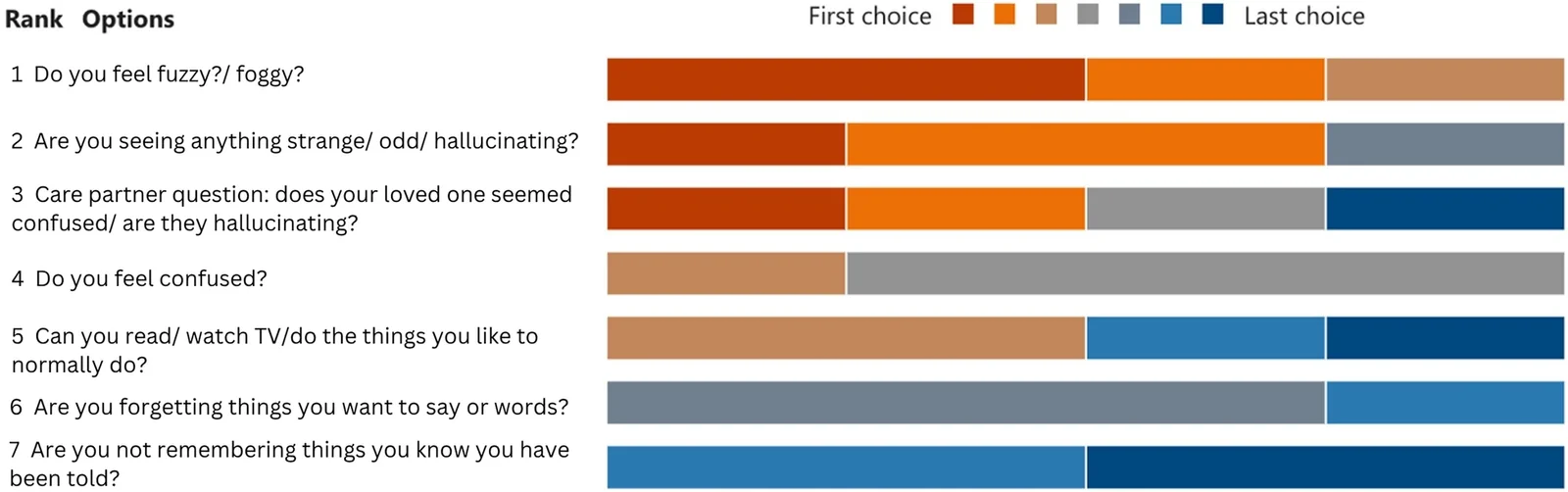
Ranking of cognitive outcome measures of early mobility in the CSICU.

**Figure 5:**
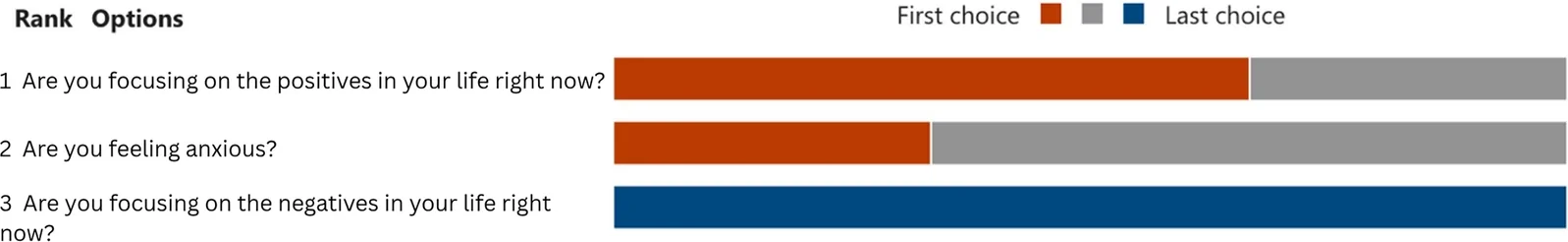
Ranking of mental outcome measures of early mobility in the CSICU.

**Figure 6:**
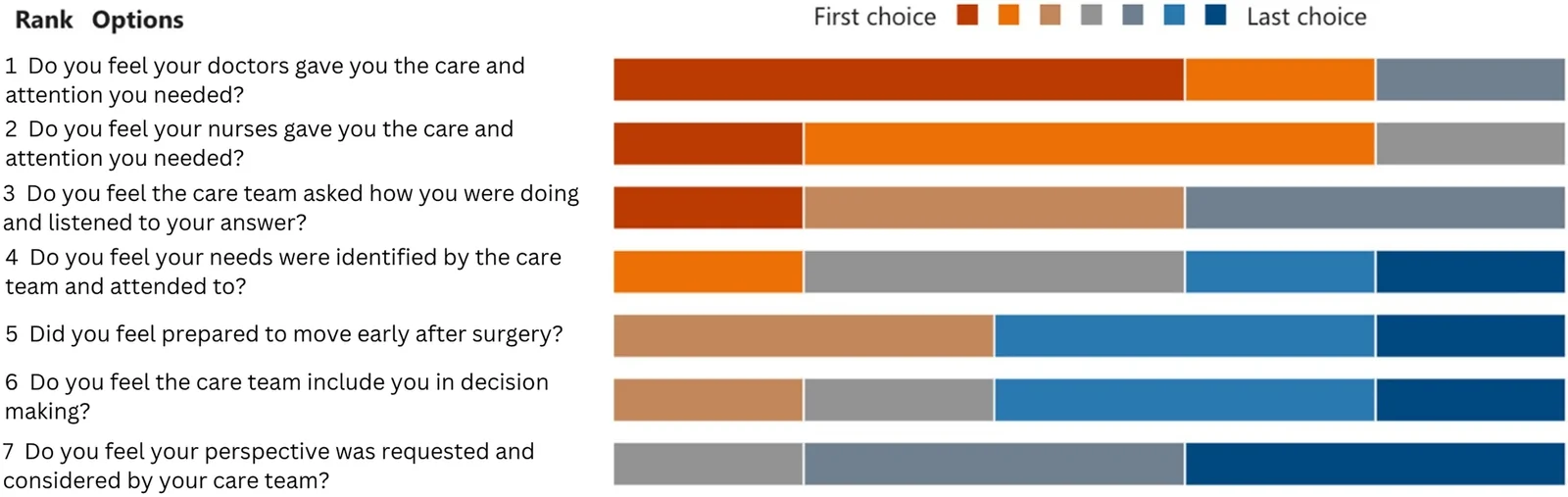
Ranking of experience measures of early mobility in the CSICU.

## 4 Discussion

Ten PWLE participated in two virtual consultation workshops, during which a definition of early mobility was co-created, and early mobility-related barriers, facilitators, outcome measures, and experience measures were generated and prioritized. The research team, including PWLE and clinician partners, then enhanced the definition to incorporate elements identified as necessary by clinicians. Barriers, facilitators, outcome measures, and experience measures were generated and prioritized by PWLE to support early mobility intervention implementation and future evaluation. Taken together, this work contributes lived experience knowledge that reconceptualizes how early mobility is defined, the barriers and facilitators to early mobility, and how early mobility interventions should be evaluated in future research. Such knowledge is important to support the integration of early mobility into CSICU clinical care.

### 4.1 Definitions

To our knowledge, this study is one of the first to co-create a definition of early mobility with PWLE and clinicians for the CSICU: *a progression of functional life activities that begins as soon as the patient can participate (typically within six hours post-surgery), where the patient uses their muscle strength intentionally.* Important elements of the definition include concepts such as *progression*, *functional life activities*, *as soon as the patient can participate*, and *intentionally*. The important elements of our co-created definition have never been included in the same definition.

*Progression* is included because mobility is not one activity but rather a variety of movements that can build on one another. One definition describes a series of movements (38), similar to our proposed *progression*. This element is missing from other located published early mobility definitions (39). Including *progression* supports the spectrum of activities that will achieve the ultimate goal of hospital discharge by viewing them as changing throughout recovery. As one PWLE co-author (AG) noted, the mobility activities undertaken when a patient first wakes after CS, such as standing, are the building blocks of those undertaken closer to discharge, when standing progresses to walking. A *progression* also supports a patient-specific approach to early mobility. Depending on the patient's baseline condition, even their first mobilization could *progress* to sitting at the side of the bed or continue along the spectrum of activities to standing or sitting in a chair (40). Thus, *progression* supports how patients need to move to accomplish the ultimate goal of discharge from the hospital in a patient-specific way.

The element of *functional life activities* in the co-created definition is reflected in some early mobility interventions (39, 41–43). This element is also included in the early mobility definition by Yang and colleagues, who state that the goal of early mobility is to “improve the mobility and functional status of critically ill patients” (38). Inclusion of *functional life activities* incorporates the conceptualization of mobility in aging research to be more than just walking or cycling to include activities required to *function*, such as bathing, eating, and brushing teeth (44, 45). Including *functional life activities* in the definition reflects consensus in the PWLE workshops that mobility should be defined as the way patients need to move to be safely discharged home. This includes being able to perform *functional life activities* as they are performed before CS. Expanding the definition of mobility to include activities such as standing at a sink to brush one's teeth instead of sitting in a chair and using a kidney basin supports these activities in a clinical setting.

*As soon as the patient can participate* defines “early” as patient-specific and allows clinicians to refer to exclusion criteria and their clinical judgement to determine when to start. Two other studies of early mobility in the CSICU have noted the element of early being based on patient-specific factors *as soon as the patient can participate* (46, 47). In one of the cited studies, early was defined as when the patient was determined to be clinically stable by the attending intensivist (46). In the other study, early was defined as when the patient had achieved hemodynamic and respiratory stability (47). Using such patient-specific factors supports individualized, patient-centred care by beginning early mobility when the patient is ready, not only post-operative time. Including *typically within six hours post-surgery* was suggested by clinician members of the research team. This is meant to provide a tangible “deadline” for clinicians, requiring them to identify criteria to opt out of mobilizing their patient. The deadline engages clinicians in planning to mobilize sooner and supports staff in understanding that decisions regarding the initiation of mobility progression should occur within this timeframe, not after. The timeframe of six hours post-surgery aligns with local guidelines (48) and is supported by the literature (40, 49). The PWLE members of the research team agreed with this addition of a post-operative timeframe. They shared that they felt that early was nebulous and difficult to define and therefore could be deemed less important than other post-operative tasks. Due to this, they agreed that it was important to clarify the concept of “early” as best we could. Through our discussions, we were able to tie the ideas of “time” and “ability” to the concept of “early” in a way that was meaningful to all team members.

*Intentionally* implies that mobility requires purposeful movement and intent to move from the patient. This element is included in the early mobility definition used by the Trial of Early Activity and Mobilization Study (TEAM) investigators (50). These researchers used the wording “patients could assist with the activity using their own muscle strength and control,” which aligns with our requirement for movements to be purposeful and with intent. This element is missing from other early mobility definitions (39). The inclusion of *intentionally* clarifies that mobility does not include non-purposeful movements and passive range of motion. A recent study by our research team noted that some healthcare providers included non-purposeful movements and passive range of motion in their early mobility definitions (51). Defining early mobility as requiring patients to move *intentionally* supports using mobility to prevent or overcome ICU-acquired weakness. ICU-acquired weakness is seen more often in patients with longer mechanical ventilation, who are less likely to be mobilized out of bed (52). The diagnosis of ICU-acquired weakness includes the strength of active muscle contraction, suggesting that mobility requires contraction, and thus *intent* from the patient. This clarifies that early mobility does not include passive range of motion. *Intentionally* also ties the definition of early mobility back to the goal of mobility, to return home and be able to function. This clarifies that early mobility does not include non-purposeful movements.

### 4.2 Barriers and facilitators

To our knowledge, many of the barriers and facilitators to early mobility in the CSICU identified in this study are novel. No identified studies included the barriers of brain “fuzziness” or “fogginess” or weakness, nor the facilitators “positive attitude” nor “receiving education about health issues interacting and causing unclear discomfort”. These are important considerations for future early mobility intervention research. PWLE are equal partners in early mobility interventions, and the interventions cannot be successful without their participation. Educating healthcare providers about the brain “fuzziness” or “fogginess” experienced will support communication. Healthcare providers can provide short, clear directions that they repeat consistently to support patients in mobilizing while feeling brain “fuzziness” or “fogginess”. Mobility aides, such as walkers, can be provided to support patients in feeling more confident when they feel weak. Patients and families can also be educated about these symptoms and reassured that it is normal and that the health care team will work with them to co-create a plan to address them. The facilitators can also be shared with patients and families to help them prepare for recovery and mobility.

The barrier “paraphernalia” (lines, tubes, etc.) has been described as a barrier in the CSICU early mobility literature (53). There are ways to overcome this barrier, such as locating lines and tubes in strategic locations that support mobility (i.e., avoiding the femoral vessels), securing lines and tubes, and planning for mobility with the patient to identify who is responsible for monitoring lines and tubes (53). In addition to these strategies, patients and their families can be made aware of the safety of moving with this paraphernalia and the low rate of inadvertent removal found in the literature (1).

The facilitator “being encouraged/ forced/ helped/ reminded” has been noted in two other CSICU early mobility studies (53, 54). This has been described as the barrier of patient refusal or lack of motivation, with the suggestion of adjusting the treatment plan with patient input (53). In the other study, patients wanted motivation about the importance of early mobility, even to the point of “nagging” (54). They felt it was the healthcare provider's role to get them moving, and this was a facilitator for them (54).

### 4.3 Outcome and experience measures

The outcome and experience measures prioritized by patients can be used in future research to align intervention evaluation with patient priorities. Currently, hospital length of stay, pulmonary function, 6 min walk test, hemodynamic measurements, heart rate variability, functional outcomes, and days to walk are the most common groups of outcome measures used in early mobility studies (39). None of the physical, cognitive, nor mental outcome measures suggested by patients are included in these outcome measures. No located study included experience measures as their primary outcomes. Our recent scoping review found that patient-reported outcome measures (PROMs) were the primary outcome measures in seven CSICU early mobility studies (39). PROMs measured functional outcomes (55, 56), quality of life (57), self-efficacy (58), and pain (59–61). While using PROMs is a step towards understanding early mobility interventions from the perspective of the PWLE (39), using outcome and experience measures identified as important to PWLE further incorporates their perspectives in intervention evaluation.

Future research is required on the outcome and experience measures. Each concept needs to be clearly defined to allow for a shared understanding. Based on the definitions, tools to evaluate the outcomes and experiences identified and prioritized by PWLE in this study need to be identified. For example, there are different definitions of delirium and different tools available to measure this outcome based on the definition used (5). This future research will support aligning the outcome measures with validated PROMs to improve assessment, identification of PICS symptoms, and patient-centred care (62).

### 4.4 Limitations

There are several limitations to this study. First, our recruitment approach lacks focus on racial and ethnic diversity, which is a limitation. Future research should focus on recruiting diverse groups in both meaningful and culturally sensitive ways that facilitate developing an understanding of the needs of PWLE of different ethnicities, rather than comparing experiences among ethnicities. Second, all PWLE had surgery at the same hospital, which limits the diversity of their healthcare experiences. This work should be replicated with PWLE from other hospitals to validate and expand the findings to support their transferability. Finally, this work does not provide guidance on specific tools to measure the identified outcomes and experiences. Future research should explore the alignment of outcome and experience measures with available tools.

## 5 Conclusion

This manuscript provides a definition of early mobility that was co-created among PWLE, clinicians, and researchers. PWLE also identified and prioritized outcome and experience measures for early mobility. This important contribution of the lived experience with early mobility in the CSICU advances the literature and will empower future research to align with the perspectives and priorities of people undergoing CS. This contribution may advance the integration of early mobility into CSICU clinical care and reduce the prevalence of adverse health outcomes, such as the cognitive, mental, and/or physical health outcomes that are seen in PICS.

## Data Availability

The data that support the findings of this study are available from the corresponding author upon reasonable request.
